# Evaluation of the physical properties of bromelain-modified biodentine for direct pulp capping

**DOI:** 10.1186/s12903-024-04863-w

**Published:** 2024-09-09

**Authors:** Paridhi Agrawal, Manoj Chandak, Aditya Patel, Jay Bhopatkar

**Affiliations:** Department of Conservative Dentistry and Endodontics, Sharad Pawar Dental College and Hospital, Datta Meghe Institute of Higher Education and Research, Wardha, Maharashtra 442107 India

**Keywords:** Bromelain, Biodentine, Compressive strength, Solubility, Radiopacity, Flow, Direct pulp capping

## Abstract

**Background:**

This study aims to evaluate the compressive strength, solubility, radiopacity, and flow of Bromelain (BR)-modified Biodentine (BD) for direct pulp capping (DPC). This is suggested to determine the impact of BR on the physical properties of BD.

**Methods:**

Eighty samples were prepared according to the ISO and ADA specifications and evaluated for compressive strength, solubility, radiopacity, and flow. The compressive strength was evaluated at 24 h and 21 days via a universal testing machine. The solubility was determined by weight loss after 24-hours immersion in deionized water. Radiopacity was assessed via X-ray with aluminum step-wedges, and flow was measured by the diameter of the discs under a standard weight. Independent sample t-tests were used to statistically assess the data. A significance level of 5% was considered.

**Results:**

The compressive strength was 41.08 ± 1.84 MPa for BD and 40.92 ± 1.80 MPa for BR + BD after 24 h, and 88.93 ± 3.39 MPa for BD and 87.92 ± 3.76 MPa for BR + BD after 21 days, with no significant differences. Solubility was slightly greater in the BR + BD (2.75 ± 0.10%) compared to BD (2.62 ± 0.25%), but not significantly different. The radiopacity was similar between BD (2.82 ± 0.11 mm) and BR + BD (2.73 ± 0.10 mm). BR + BD resulted in significantly greater flow (9.99 ± 0.18 mm) than did BD (9.65 ± 0.27 mm) (*p* ≤ 0.05).

**Conclusion:**

BR-modified BD maintains BD’s physical properties, with improved flow, making it a promising DPC agent that warrants further study.

## Introduction

Dental caries induce pulpal inflammation through bacterial invasion, and even after the completion of dental procedures, pulpal tissue may persist in an inflamed state, potentially leading to chronic, low-grade inflammation and delayed healing [1, 2]. The dentinal tubules allow microorganisms to infect dental pulp tissue [3]. Bacteria, mostly gram-negative bacteria, are frequently observed in cases of pulpitis and deep caries. One of their main virulence factors is the introduction of lipopolysaccharides (LPS) into the cell wall structure [4]. LPS stimulates immune cells by binding to Toll-like receptor 4 (TLR4), activating the mitogen-activated protein kinase (MAPK) or nuclear factor kappa-B (NF-κB) pathway, and producing proinflammatory cytokines such as tumor necrosis factor (TNF)-α and interleukins (ILs)-1β, -6, and − 8 [5, 6].

In such cases, direct pulp capping (DPC) is an effective treatment. This involves the application of a biomaterial to exposed dental pulp after caries excavation to promote mineralized tissue formation and preserve pulp vitality [7]. The removal of pathogenic bacteria reduces inflammation, allowing the pulpal immune system to neutralize intratubular diffusing substances and decrease proinflammatory cytokines [8]. This reduction in inflammation leads to the healing of the pulpal tissue and the formation of a reparative dentin bridge or mineralized tissue barrier [1].

Selecting the best biomaterial for every clinical situation is difficult because of the abundance of biomaterials available for DPC [9]. Good handling properties, radiopacity, bioactivity, ability to form a good seal, to be insoluble in tissue fluids and to promote the formation of a mineralized tissue barrier, are considered essential qualities of ideal DPC materials [10].

Given the exposure to masticatory stresses and condensation pressure, the compressive strength of materials is crucial for ensuring longevity in terms of placement and functionality [11]. The solubility of restorative material is a critical consideration for DPC. While less solubility is preferred for prolonged sealing and durability, some solubility is necessary to encourage mineralization close to the vital tissue despite preserving an efficient seal [12]. According to the International Organization for Standardization (ISO) guidelines, materials must possess a radiopacity of 3 mm of aluminum to be distinguishable from dentin [13]. Radiopacity is vital for quality assessment. Flow and adherence are crucial factors when a material is selected. High flowability facilitates adhesion to dentin, allowing penetration into dentinal tubules. This promotes an efficient seal and the formation of a dentin bridge, minimizes contamination, and intercepts microleakage — all pivotal aspects of effective vital pulp therapy [14].

Despite the advancements in DPC materials and the availability of various options, including zinc oxide eugenol, glass ionomer cement, resin-modified glass ionomer cement, calcium hydroxide (CH), light-cured CH materials, mineral trioxide aggregate, Biodentine (BD), bioaggregates, and bioceramics, no material has yet achieved ideal characteristics for DPC procedures [15].

BD, which was introduced as a bioactive dentine substitute in 2011, is a unique cement that mostly consists of tricalcium silicate (TCS) and has extraordinary bioactive properties [16]. Unlike mineral trioxide aggregate, which contains inorganic compounds such as calcium sulfate and aluminate and bismuth oxide, BD’s main components are pure TCS, calcium carbonate, and a radiopacifier, zirconium oxide [17, 18]. Compared with mineral trioxide aggregate, BD has better mechanical characteristics, improved color stability, a faster initial setting time (10–12 min), and an easier application procedure [19–22].

Bromelain (BR) is a mixture of enzymes taken from the fruits and stems of pineapples (Ananas comosus). This mixture contains a variety of enzymes, such as cellulase, phosphatase, glucosidase, peroxidase, and thiol endopeptidases, among several protease inhibitors [23, 24]. BR has shown numerous therapeutic effects, such as anti-inflammatory, antibacterial, and anticancer effects, although its exact mechanism remains unclear [25]. BR stimulates inflammatory mediators such as IL-1β, IL-6, INF-γ, and TNF-α [26, 27]. In contrast, BR has the ability to decrease the secretion of granulocyte colony-stimulating factor (G-CSF), IL-1β, TNF-α, and IL-6 under inflammatory conditions in which immune cells are stimulated [28]. BR reduces IκB phosphorylation, preventing NF-κB activation in human cancer cells [29], and inhibits MAPK pathway activity in RAW264.7 cells [30]. Furthermore, bromelain exhibits antibacterial action against *Porphyromonas gingivalis* and *Enterococcus faecalis* [31] and reduces edema after third molar extraction [32]. It also inhibits inflammatory cytokines in human dental pulp cells (hDPCs) treated with LPS, and stimulates mineralized calcium nodule formation and alkaline phosphatase (ALP) activity [33]. Thus, BR has anti-inflammatory effects, and its ability to mineralize suggests its potential in dentinogenesis.

By harnessing this mineralization potential and the anti-inflammatory and antimicrobial effects of BR, it was speculated that BR would have synergistic effects on BD, increasing its potential as a DPC agent. Before utilizing this combination of BR and BD for direct pulp capping, it is important to study the effects of BR on the physical properties of BD. Hence, the present study was carried out to evaluate and compare the compressive strength, solubility, radiopacity, and flow of BR-modified BD as a direct pulp capping material. The null hypothesis tested was that there would be no significant effect on the physical properties of BD after the addition of BR.

## Materials and methods

This experimental investigation was approved by the institutional ethical committee (Reference number- DMIMS(DU)/IEC/2022/768. Open Epi software (version 3.04.04) was used to calculate the sample size for the study performed by Kaup M et al. [12]. The calculation was performed with 80% power and a 95% confidence interval, taking into account the mean solubility of BD in distilled water. The per group sample size was determined to be 9 (approximately 10 per group).

### Sample grouping

**Fig. 1:**
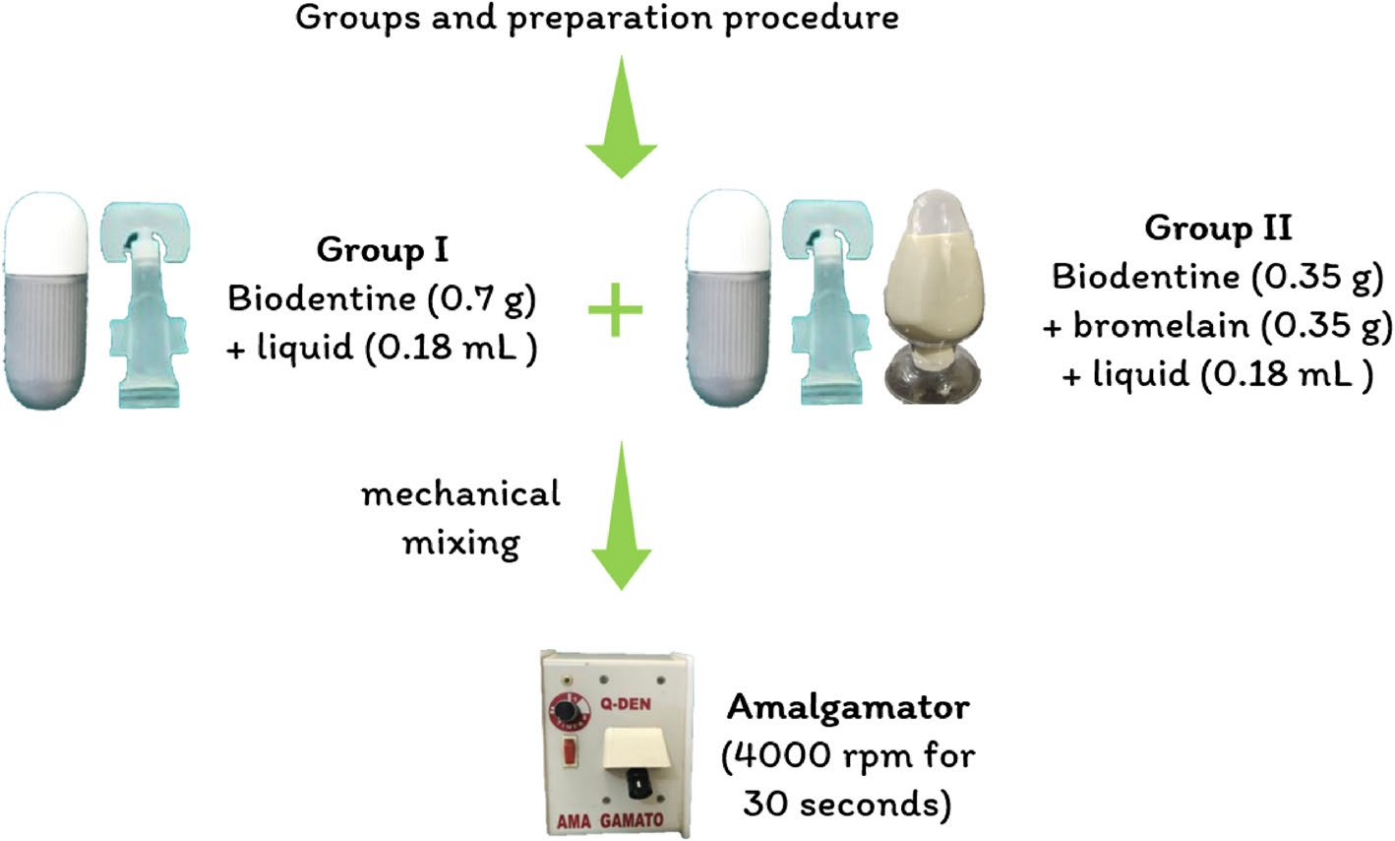
Preparation procedure of the specimens

The samples were grouped into two groups: Group I (Biodentine) and Group II (Bromelain + Biodentine). The details of the groups are shown in Table 1 and Fig. 1

**Table 1:** Details of the groups and preparation procedure

Group	Manufacturer details	Composition	Preparation procedure
**Group I-** **Biodentine (BD)**	Septodont, France (Lot No.- 828038)	Powder- Tricalcium silicate (80.1%), calcium carbonate (14.9%), zirconium oxide (5%)	0.7 g (1 capsule) of powder mixed mechanically with 0.18 mL liquid in an amalgamator at speed of 4000 revolutions per minute for 30 s as shown in Fig. 1.
		Liquid- aqueous mixture of hydrosoluble polymer and calcium chloride	
**Group II-** **Combination of Bromelain with Biodentine** **(BR + BD)**	Brisk bioscience, India (Lot No.- KBDG/BR/071022)	Equal proportions of BR and BD (50:50)	0.35 g of BR and 0.35 g of BD mixed mechanically with 0.18 mL liquid in an amalgamator at speed of 4000 revolutions per minute for 30 s as shown in Fig. 1.

### Compressive strength test

#### Sample preparation

**Fig. 2:**
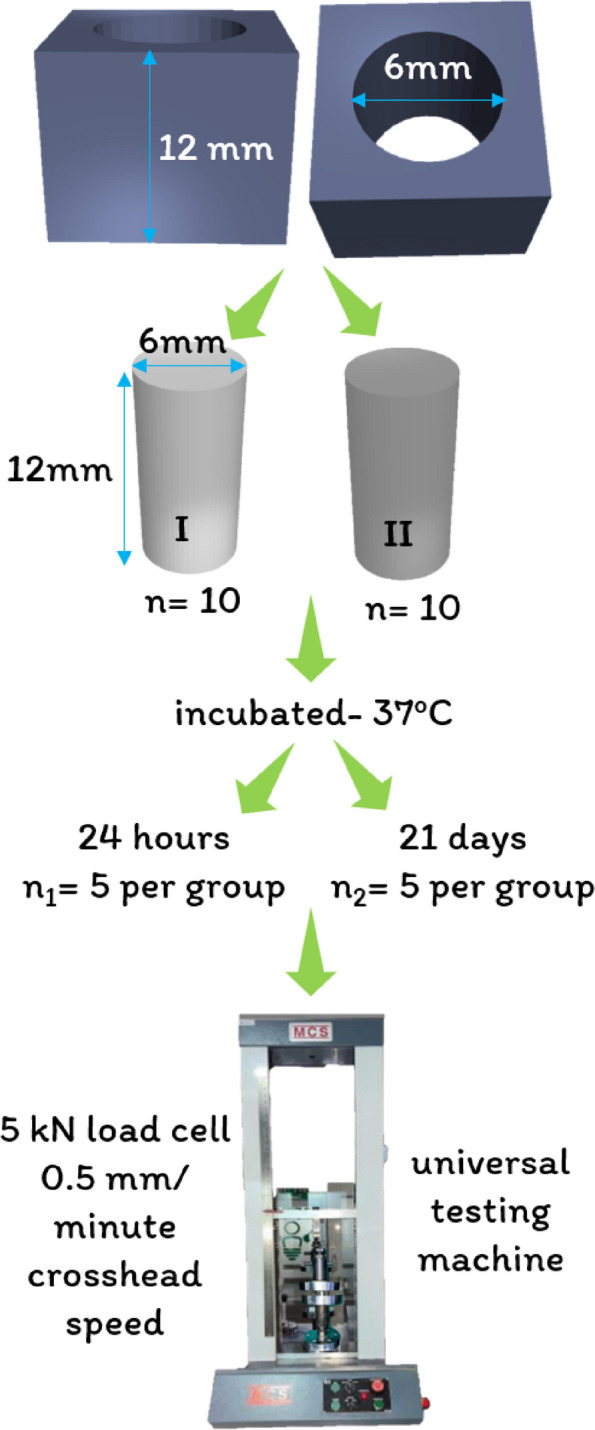
Compressive strength testing protocol

The compressive strength of the specimens was assessed in accordance with ISO 6876:2012 guidelines [34]. As shown in Fig. 2, a stainless steel mold with a height of 12 mm and diameter of 6 mm was fabricated. Twenty cylindrical samples (*n* = 10 per group) were prepared for Group I (BD) and Group II (BR + BD) by filling the test materials in a stainless steel mold. The samples were then demolded, and their flat tops were smoothed using silicone-carbide abrasive paper with a grit size of 600 to obtain a standardized sample with a height of 12 mm.

#### Sample testing

The samples were then divided into two halves for each group (*n* = 5) and incubated at 37 °C. Half of the samples were incubated for 24 h, and the other half were incubated for 21 days. After 24 h and 21 days, the samples were compressed with a 5 kN load cell at a 0.5 mm per minute crosshead speed between the platens of the universal testing machine (MCS Mild Steel Computerized Universal Testing Machine, Maharashtra, India) until failure. The data thus obtained were the maximum perpendicular loading force (N) the specimen could withstand. The compressive strength (MPa or N/mm^2^) of the specimens was calculated using the following formula:

$$\mathrm{Compressive}\;\mathrm{strength}\:=\:4\mathrm F/\mathrm d^2$$

where F = the maximum perpendicular loading force in newtons (N).

d = the diameter of the specimen in mm (6 mm).

### Solubility

#### Sample preparation

**Fig. 3:**
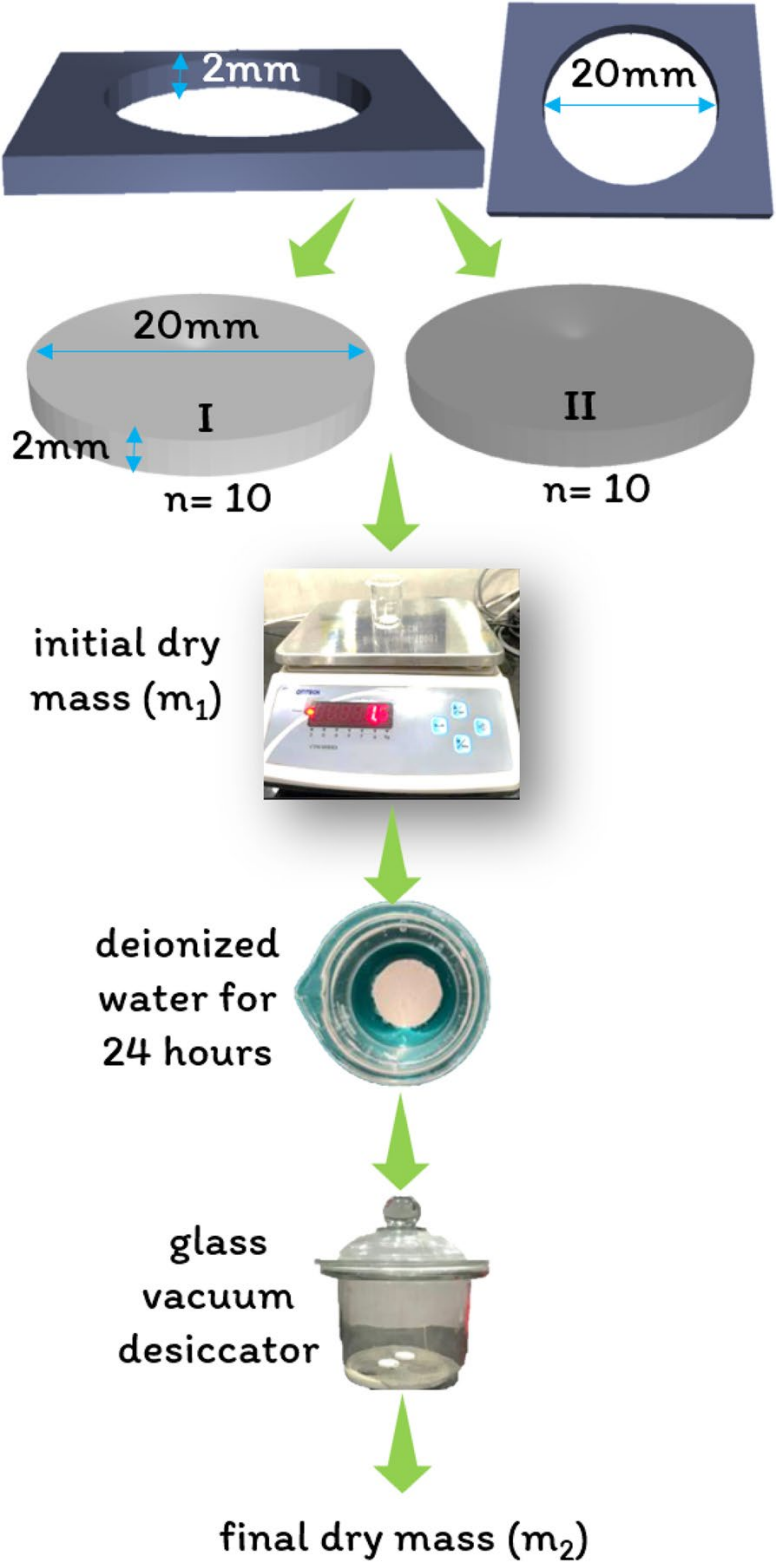
Solubility testing protocol

The solubility of the specimens was assessed in accordance with ISO 6876:2012 guidelines [34] and ANSI/ADA specification number 57/2000 [35]. As shown in Fig. 3, a stainless-steel mold with a height of 2 mm and diameter of 20 mm was fabricated. Twenty cylindrical samples (*n* = 10 per group) were prepared for Group I (BD) and Group II (BR + BD) by filling the test materials in a stainless steel mold. The samples were then demolded after the completion of their setting time.

### Sample testing

The initial dry mass (m_1_) of the samples was recorded using a digital weight balance (CTM-10001 Contech, Maharastra, India). Each of the samples was then immersed for 24 h in glass beakers filled with 25 mL of deionized water at 37 °C. After 24 h, the samples were removed from the deionized water and placed in a glass vacuum desiccator (Cole-Parmer, Maharashtra, India). Thereafter, the final dry weight of the specimens (m_2_) was measured. The 24-hour solubility in distilled water (% weight loss) of the specimens was calculated using the following formula:

$$\mathrm{Solubility}=\left[\left({\mathrm m}_1-{\mathrm m}_2\right)/{\mathrm m}_2\right]\times100$$

where m_1_ = initial weight of the sample (in grams).

m_2_ = final dry weight of the sample (in grams).

### Radiopacity

#### Sample preparation

**Fig. 4:**
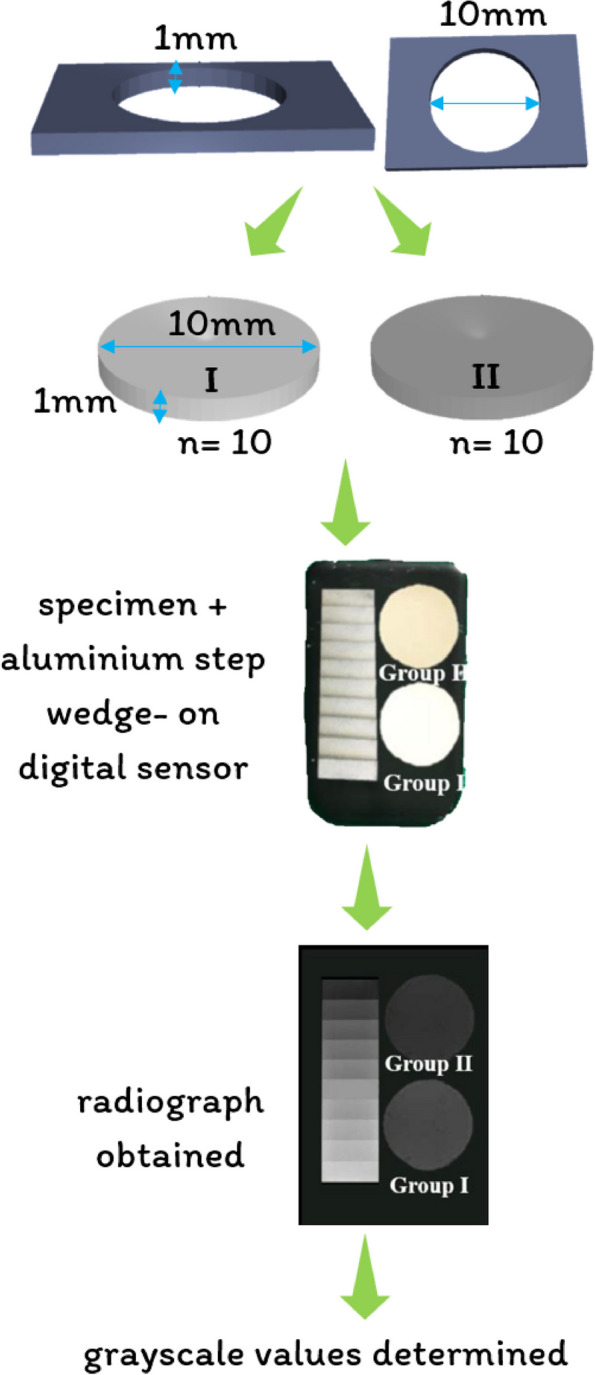
Radiopacity testing protocol

The specimens’ radiopacity was assessed in accordance with ISO 6876:2012 guidelines [34] and ANSI/ADA specification number 57/2000 [35]. As shown in Fig. 4, a stainless-steel mold with a height of 1 mm and diameter of 10 mm was fabricated. Twenty cylindrical samples (*n* = 10 per group) were prepared for Group I (BD) and Group II (BR + BD) by filling the test materials in a stainless steel mold. The samples were then demolded after the completion of their setting time.

#### Sample testing

- Specimens with aluminum step wedges (Matcon, Netherlands, Europe) were placed over a digital sensor (Eco Smart RVG, Confident Sales India Private Limited, Banglore, India). The X-ray beam was fixed at an object and source distance of 320 mm, with zero degrees of horizontal and vertical angulations. An X-ray unit with an exposure time of 0.20 s operating at 7 mA and 65 kVp was used, and a digital radiograph was obtained. The images thus obtained were assessed using ImageJ software (ImageJ, Digimizer Image Analyzing Software, MedCalc Software Limited, Belgium), and the grayscale values were determined. The radiopacity in mm of aluminum thickness was determined using the following conversion formula [36]. Radiopacity in mm of aluminum thickness = (A x 2/B) + C.

where A = grayscale value of material – grayscale value of aluminum.

step-wedge increment immediately below the material.

B = grayscale value of aluminum step-wedge increment.

immediately above the material - grayscale value of aluminum.

step-wedge increment immediately below the material.

C = mm of aluminum thickness immediately below the material.

### Flow

**Fig. 5:**
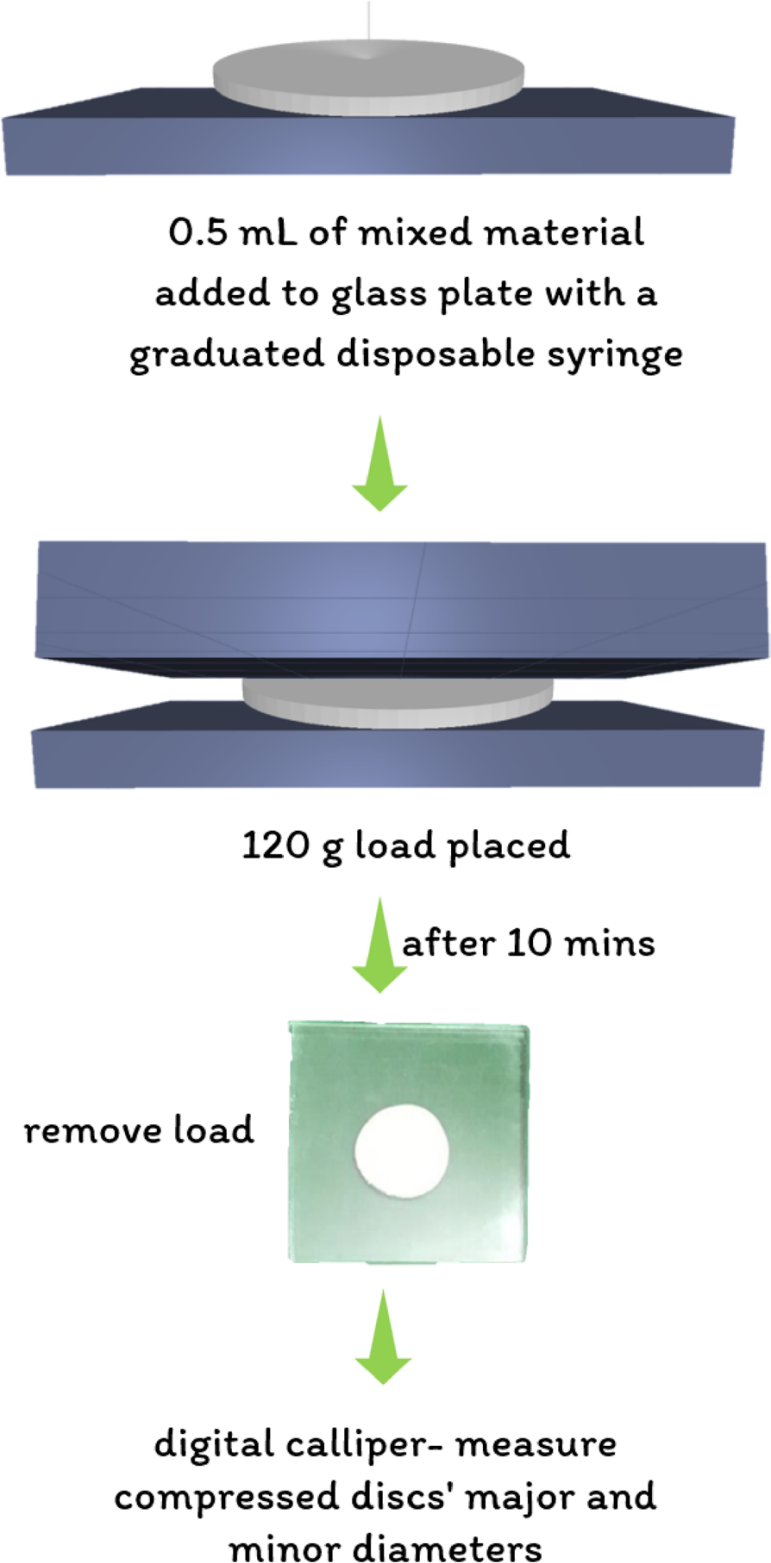
Flow testing protocol

- The specimen flow was assessed in accordance with ISO 6876:2012 guidelines [34]. As shown in Fig. 5, 0.5 mL of the mixed material was added to a glass plate utilizing a 3 mL graduated disposable syringe. After 10 min, a second glass plate with a weight of 20 g and a load of 100 g was subsequently added to the top of the material. The load was removed after ten minutes of mixing, and a digital caliper was used to measure the average major and minor diameters of the compressed discs.

### Statistical analysis

Statistical analysis was performed with SPSS version 21 software after data compilation. Descriptive statistics are presented as the mean, standard deviation, frequency, and percentage. The normality of the data distribution was assessed through Kolmogorov-Smirnov and Shapiro-Wilk tests, confirming adherence to a normal distribution. Subsequently, independent sample t tests were applied to determine statistically significant differences between Group 1 (BD) and Group 2 (BR + BD). The level of statistical significance was set at *p* ≤ 0.05.

## Results

### Compressive strength results

**Fig. 6:**
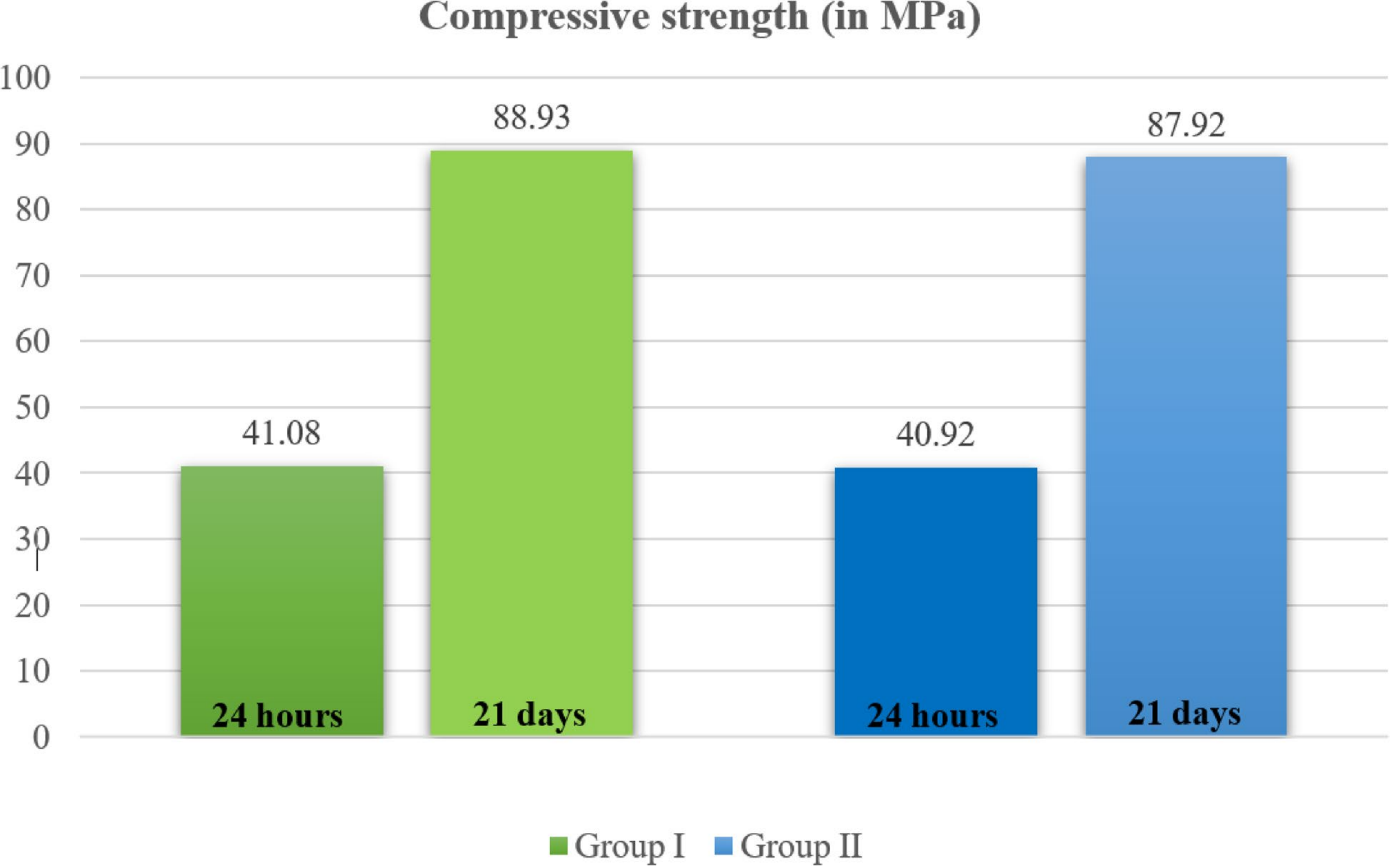
Compressive strength after 24 h and 21 days for Group I (BD) and Group II (BR combined with BD)

As shown in Table 2 and Fig. 6, the mean compressive strength after 24 h was 41.082 ± 1.84 MPa for Group I (BD) and 40.92 ± 1.80 MPa for Group II (BR combined with BD). Similarly, the mean compressive strength after 21 days was 88.93 ± 3.39 MPa for Group I (BD) and 87.92 ± 3.76 MPa for Group II (BR combined with BD). Although the compressive strength after 24 h and 21 days was marginally greater for Group I (BD) than for Group II (BR combined with BD), the difference was not statistically significant.

### Solubility results

**Fig. 7:**
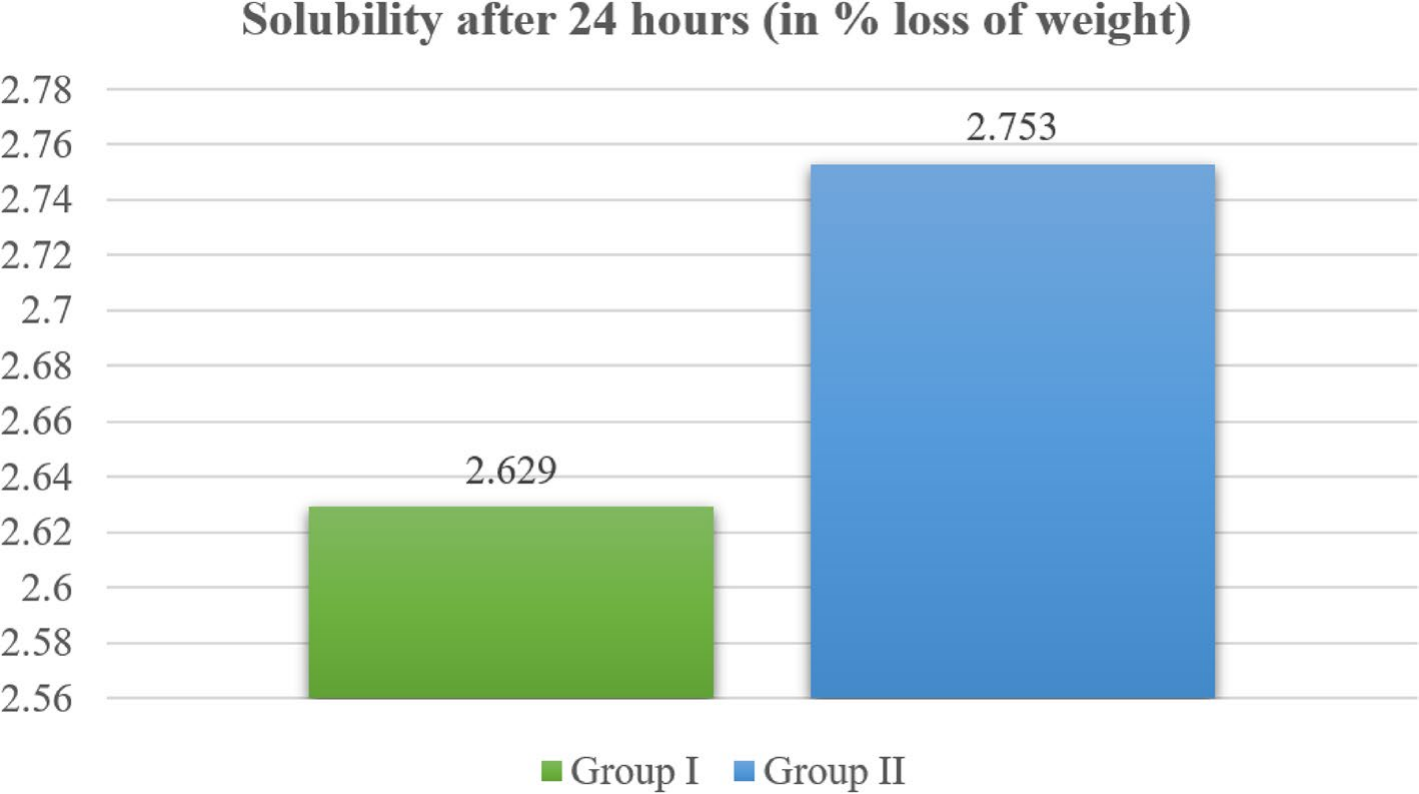
Solubility after 24 h for Group I (BD) and Group II (BR combined with BD)

As shown in Table 2 and Fig. 7, the mean solubility after 24 h was 2.62 ± 0.25% for Group I (BD) and 2.75 ± 0.10% for Group II (BR combined with BD), suggesting that the solubility was slightly elevated in Group II (BR combined with BD) compared with Group I (BD); however, there was no statistically significant difference between them.

### Radiopacity results

**Fig. 8:**
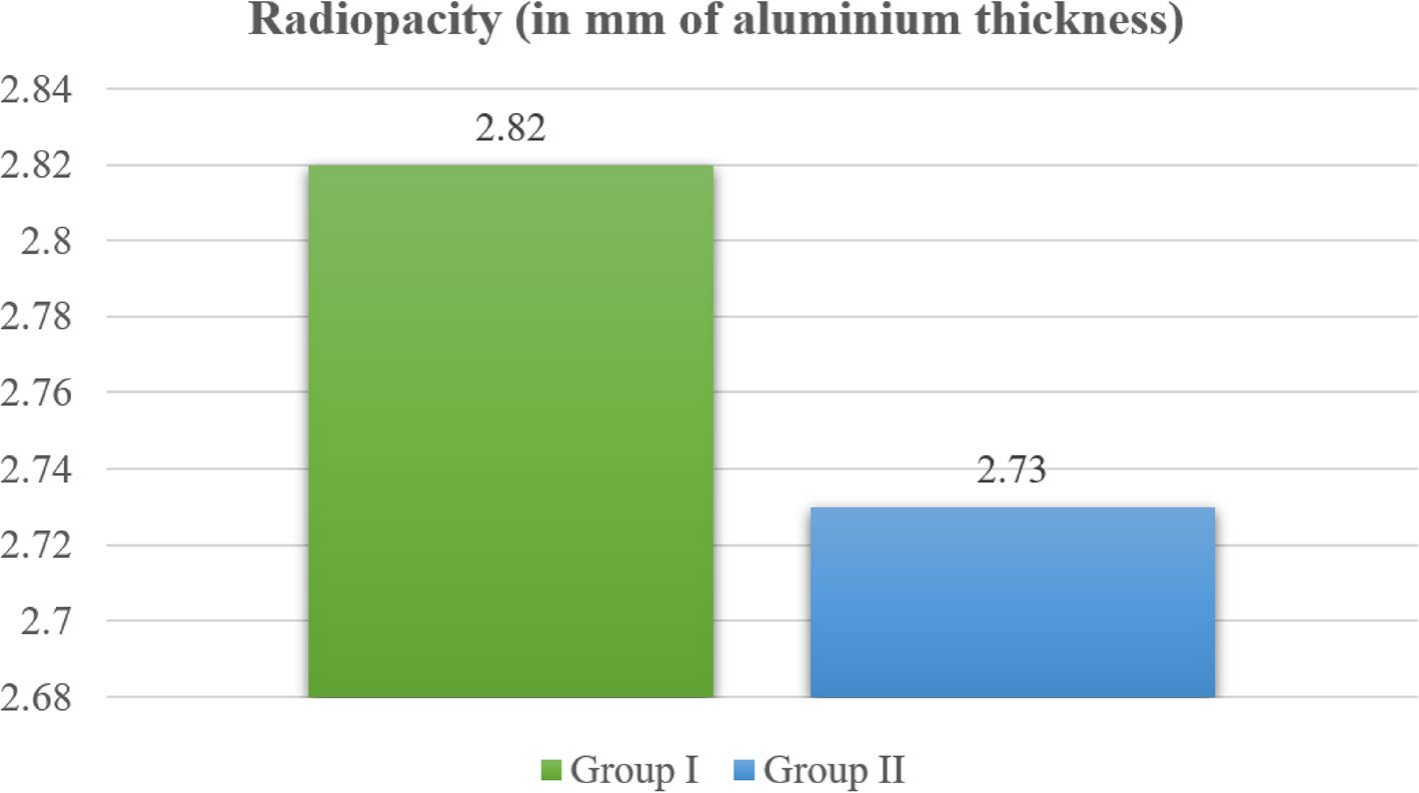
Radiopacity in Group I (BD) and Group II (BR combined with BD)

As shown in Table 2 and Fig. 8, the mean radiopacity for Group I (BD) measured 2.82 ± 0.11 mm of aluminum, whereas for Group II (BR combined with BD), it was 2.73 ± 0.10 mm of aluminum, but there was no statistically significant difference between them.

### Flow results

**Fig. 9:**
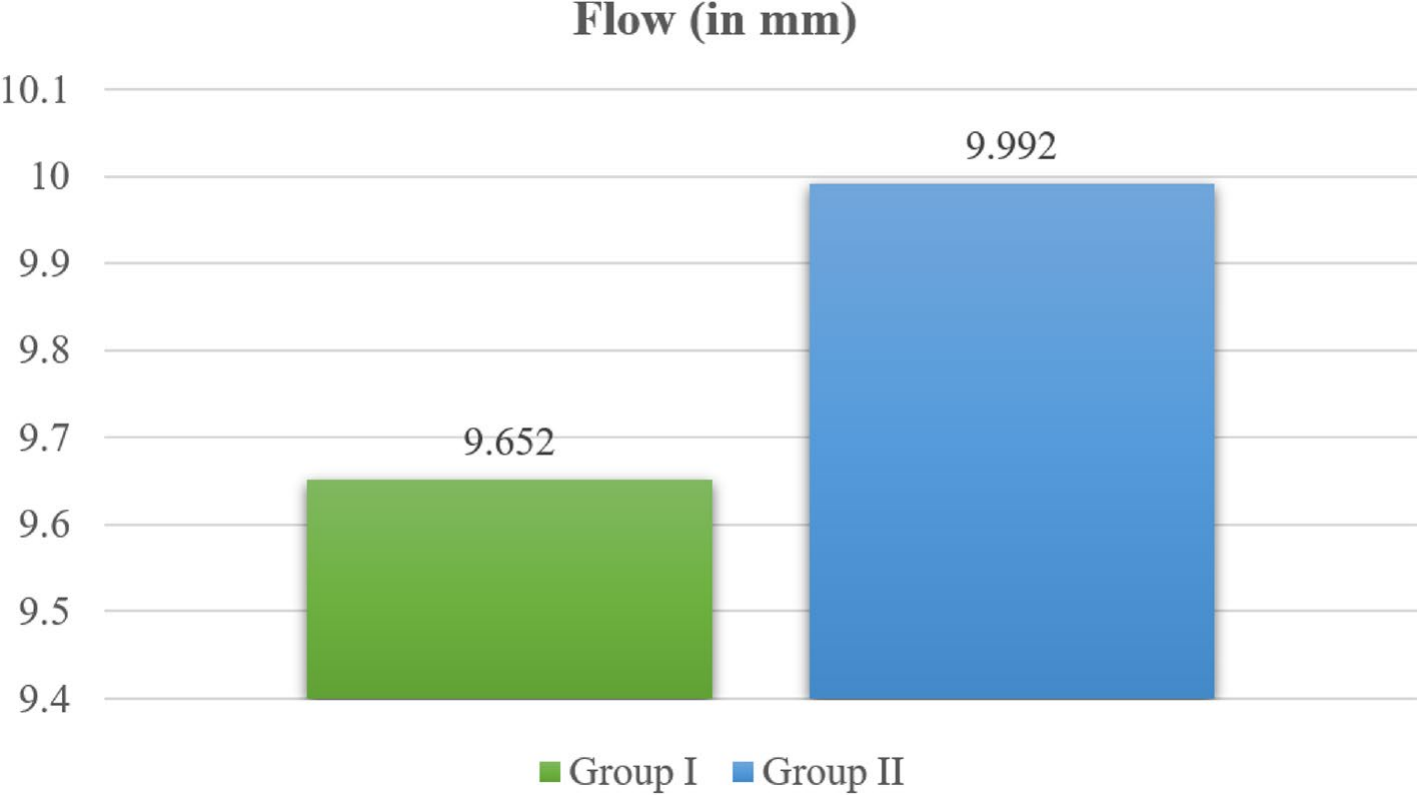
Flow in Group I (BD) and Group II (BR combined with BD)

As shown in Table 2 and Fig. 9, the mean flow in Group I (BD) was 9.65 ± 0.27 mm, whereas in Group II (BR combined with BD), it was 9.99 ± 0.18 mm. Here, Group II (BR combined with BD) presented a significantly greater flow than did Group I (BD) (*p* ≤ 0.05).

**Table 2:** Descriptive statistics of Group I (BD) and Group II (BR combined with BD)

Physical properties	Groups	Mean	Standard Deviation	Mean Difference	*t* value	*p* value	95% Confidence interval	
							Upper Bound	Lower Bound
Compressive Strength after 24 h (MPa)	Group I	41.082	1.848	0.159	0.195	0.848	-1.55738	1.8753
	Group II	40.923	1.804					
Compressive Strength after 21 days (MPa)	Group I	88.936	3.391	1.013	0.632	0.535	-2.35424	4.3802
	Group II	87.923	3.766					
Solubility after 24 h (MPa)	Group I	2.629	0.258	-0.124	-1.411	0.184	-0.31579	0.067
	Group II	2.753	0.102					
Radiopacity (mm of aluminum thickness)	Group I	2.820	0.115	0.090	1.825	0.085	-0.01359	0.1935
	Group II	2.730	0.105					
Flow after 10 min (mm)	Group I	9.652	0.279	-0.340	-3.181	0.005*	-0.56457	-0.115
	Group II	9.992	0.189					

*Statistical significance was defined as *p* ≤ 0.05

Where Group I is Biodentine (BD)

Group II is Bromelain (BR) combined with Biodentine (BD)

## Discussion

Selecting the best biomaterial for DPC is challenging due to the abundance of options, each requiring essential qualities such as adequate compressive strength, solubility, flow, and radiopacity [10].

Although BD has better mechanical characteristics, improved color stability, faster initial setting time, and easier application [19–22], it has limitations in terms of consistency and anti-inflammatory and antimicrobial effects [37, 38]. BR possesses anti-inflammatory and antibacterial effects [30, 32]. In addition, in inflamed human dental pulp cells, BR inhibits the production of inflammatory cytokines while stimulating mineralized calcium nodules and ALP activity, showing anti-inflammatory effects and mineralization potential and leading to dentinogenesis [33]. Considering these properties of BR, it was combined with BD, and its effect on the physical properties of BD was studied.

The compressive strength serves as a guide for the material’s strength [22]. A cement with higher compressive strength can be densely packed into the cavity, ensuring a tight seal and enhancing the overall prognosis of the restoration. Adequate compressive strength is vital for capping materials to withstand masticatory forces effectively [39]. The compressive strength of the materials was evaluated by compressing them between the plates of a universal testing machine. For this purpose, a height-to-diameter ratio of 2 was chosen, and cylindrical specimens with a height of 12 mm and diameter of 6 mm were fabricated. This ratio helps prevent the restraining effects of the loading plates. Ratios exceeding 2 may result in specimen bulking, whereas lower ratios necessitate the application of a correction factor for evaluating the compressive strength [40]. In our study, there was no statistically significant difference in the compressive strength values for either group, suggesting that the addition of BR to BD does not impact its compressive strength. Additionally, the results for Group I (BD) were congruent with the findings of Natale LC et al. (2015) [41], Al-Sherbiny IM et al. (2021) [39], and Jang YE et al. (2014) [42]. This compressive strength of BD is attributed to the presence of a hydrosoluble polymer in BD, which reduces the water-powder ratio, increases the density of calcium carbonate, and results in a smooth structure composed of fine particle agglomerates of calcium silicate hydrate (CSH) gel hydration products, which contribute to strong interparticle adhesion.

For a direct pulp capping material, less solubility is preferred for prolonged sealing and durability, but some solubility is necessary to encourage mineralization close to the vital tissue despite preserving an efficient seal [12]. The release of Ca and hydroxyl ions is important and is related to the solubility of the material [43]. Additionally, higher solubility results in more gaps and voids in the material, paving the way for the ingress of microorganisms [44]. The solubility test in this study adhered to ISO 6876:2012 guidelines [34] and ANSI/ADA specification number 57/2000 [35], wherein the reduction in mass (loss of weight) after storage in deionized water was calculated. The results are expressed as a percentage of the original weight of the specimen. According to ISO 6876:2012 [34], the solubility of an ideal material in terms of weight loss should be less than 3% after 24 h. The results of the present study indicate no significant difference in the solubilities of the two groups. With a solubility close to 3%, the addition of BR to BD does not affect the solubility of BD. Similar results to those of Group I (BD) were obtained by Gandolfi MG et al. (2015) [43], Kaup M et al. (2015) [12], Ashi T et al. (2022) [45], and Rabello CZ et al. (2022) [46]. This could be attributed to the limited quantity of dispersed polycarboxylic ether-based water reducers that superplasticize the mixing fluid and to the calcium chloride water reducing agent present in the liquid of BD.

Direct pulp capping materials must exhibit radiopacity for effective evaluation of the quality of restoration, and materials with lower radiopacity may be indistinguishable from dentin [46]. The thickness and molecular weight of a material impact its radiopacity [40]. The radiopacity of an ideal material must be sufficient to support its chemical and physical properties [47]. The radiopacity of the specimens was assessed in accordance with ISO 6876:2012 guidelines [34] and ANSI/ADA specification number 57/2000 [35] using digital radiography. According to these standards, for a 1 mm thick sample, a radiopacity of more than 3 mm aluminum thickness is needed. The radiopacity of both groups was less than 3 mm aluminum, with no significant difference between them, suggesting that the addition of BR to BD does not impact its radiopacity. The findings for Group I (BD) were consistent with the findings of Kaup M et al. (2015) [12], Lucas CP et al. (2017) [40], Ochoa-Rodriguez VM et al. (2018) [48], and Sen HG et al. (2023) [47]. This could be attributed to the 5% zirconium oxide present in BD, which has a low radiopacity due to the lower atomic number of zirconia and is not enough to provide adequate radiopacity to BD with a thickness of more than 3 mm.

Good material flow is essential because it enhances adhesion to dentin, facilitating penetration into dentinal tubules. This, in turn, promotes an effective seal, the formation of a dentin bridge, and minimizes contamination while intercepting potential issues such as microleakage [49]. The assessment of flow in this study adhered to ISO 6876:2012 guidelines [34] and ANSI/ADA specification number 57/2000 [35]. In the present study, the addition of BR to BD significantly increased its flow. Additionally, the findings of Group I (BD) were reinforced by the findings of Torres FFE et al. (2020) [49], Carvalho FM et al. (2021) [50], and Pelepenko LE et al. (2021) [51]. This can be attributed to the polycarboxylate present in BD, which acts as a plasticizer.

The limitations include that in clinical scenarios, the effectiveness of these direct pulp capping materials may be influenced by the presence of local factors, which was not considered in the present study. More studies are needed to assess their biological properties, rheological properties and other mechanical properties in addition to their compressive strength, solubility, radiopacity and flow. Future studies are recommended to investigate the possible effects of combining BR with BD at ratios other than 1:1. Microstructural examinations and evaluation of the cytotoxicity of modified BDs are also recommended.

## Conclusion

The present study highlights the potential of combining BD with BR for DPC. The comparable compressive strength and solubility between the two groups suggest similar mechanical properties and durability. Additionally, the radiopacity results indicate that the addition of BR did not affect the radiopacity of BD. Notably, the significantly greater flow of the BR with BD suggests improved clinical handling characteristics.

### Clinical significance

The addition of BR does not alter the physical properties of BD, making BD with BR a promising option for DPC. BR’s mineralization, anti-inflammatory, and antimicrobial effects could further enhance the performance of BD in pulp therapy.

## Data Availability

The data that support the findings of this study are available from the corresponding author upon reasonable request.

## Abbreviations

ADA: American Dental Association
ANSI: American National Standards Institute
ALP: alkaline phosphatase
BD: Biodentine
BR: bromelain
CH: calcium hydroxide
CSH: calcium silicate hydrate
DPC: direct pulp capping
G-CSF: granulocyte colony-stimulating factor
TCS: tricalcium silicate
LPS: lipopolysaccharides
TLR: Toll-like receptor
TNF: tumor necrosis factor
IL: interleukin
ISO: International Organization for Standardization
MAPK: mitogen-activated protein kinase
MPa: megapascal
MM: millimeters
NF-κB: nuclear factor kappa-B
kN: kilo newton
